# Endometriosis Malignant Transformation: Epigenetics as a Probable Mechanism in Ovarian Tumorigenesis

**DOI:** 10.1155/2018/1465348

**Published:** 2018-03-27

**Authors:** Jiaxing He, Weiqin Chang, Chunyang Feng, Manhua Cui, Tianmin Xu

**Affiliations:** The Second Hospital of Jilin University, Jilin, Changchun 130041, China

## Abstract

Endometriosis, defined as the presence of ectopic endometrial glands and stroma outside the uterine cavity, is a chronic, hormone-dependent gynecologic disease affecting millions of women across the world, with symptoms including chronic pelvic pain, dysmenorrhea, dyspareunia, dysuria, and subfertility. In addition, there is well-established evidence that, although endometriosis is considered benign, it is associated with an increased risk of malignant transformation, with the involvement of various mechanisms of development. More and more evidence reveals an important contribution of epigenetic modification not only in endometriosis but also in mechanisms of endometriosis malignant transformation, including DNA methylation and demethylation, histone modifications, and miRNA aberrant expressions. In this present review, we mainly summarize the research progress about the current knowledge regarding the epigenetic modifications of the relations between endometriosis malignant transformation and ovarian cancer in an effort to identify some risk factors probably associated with ectopic endometrium transformation.

## 1. Introduction

Endometriosis is a chronic and hormone-dependent disease, defined as the presence of ectopic endometrial glands and stroma outside the uterine cavity [1]. The prevalence of endometriosis is widely different for various ethnic groups [2–4], which is likely to be about 5–15% of reproductive-age women and 3 to 5% of postmenopausal women [3]. To date, it is well established that endometriosis is a chronic inflammatory disease and the chronic inflammation is associated with pain and infertility [1]. As a common disease in reproductive woman, ectopic endometrium is predominantly detected in the pelvic compartment like the utero-sacral ligament, Douglas cavity, and ovary; moreover, the ectopic endometrial tissue can attach to other tissues including the bladder and ureter as well as the lung. Though several hypotheses are reported in order to explain the pathogenesis of endometriosis, mainly including coelomic metaplasia, retrograde menstruation, and lymphatic and vascular dissemination, none of them can explain all the different types of endometriosis.

Despite the fact that endometriosis is considered a benign condition because of its normal histology, the cellular, histologic, and molecular data strongly demonstrate that endometriosis has neoplastic characteristics [5, 6]. There is strong evidence that endometriosis shares striking features with malignancy [5]. Similar to cancer, ectopic endometrial tissue can result in normal tissue dissemination, invasion, and organ damage, as well as neoangiogenesis. It is reported that endometriosis is associated with ovarian cancer in all aspects of research fields including epigenetics; the link between endometriosis and ovarian cancer was reported for the first time as early as 1925 [7]. In the last nine decades, epidemiological investigation has been accumulated that endometriosis may contribute to the development and progression of ovarian cancer. In a cohort study by Melin et al., where 63,630 eligible women diagnosed with endometriosis entered, the risk of ovarian cancer (SIR 1.37) was moderately increased as compared with that of the general population [8]. As technology develops, multiple mechanisms about the occurrence of ovarian cancers associated with malignant transformation of endometriosis have been studied for a long time, but they still remain elusive. Currently, it is well demonstrated that epigenetic modifications contribute to ovarian tumorigenesis. Epigenetics is described as a heritable modification in gene expression without alteration of DNA sequence compared with gene mutation [9]. The epigenetic modifications so far involve DNA methylation, histone modifications, and noncoding microRNAs (miRNAs) [10–12]. In the present review, we mainly summarize the research progress regarding the epigenetic modifications of the relations between endometriosis malignant transformation and ovarian cancer in an effort to identify some risk factors probably associated with ectopic endometrium transformation.

## 2. DNA Methylation

DNA methylation, the most frequently studied epigenetic alteration, occurs at the carbon-5 position of cytosine residues, exclusively in CpG dinucleotide sequences, and inhibits gene transcription [13]. DNA methylation, referring to the addition of the methyl groups into the cytosines from *S*-adenosyl L-methionine, is mediated by a family of enzymes known as the DNA methyltransferases (DNMTs) including DNMT1, DNMT3a, and DNMT3b. DNA methylation is a heritable epigenetic occurrence that significantly regulates gene expression without changing DNA sequence [14]. Most CpG sites in the human genome are methylated. However, local CpG islands, the CpG-rich regions, founded in the promoter regions of widely expressed genes are in unmethylated conditions [15, 16]. It is well evidenced that hypermethylation of genes can result in inhibition of gene expression, whilst hypomethylation may give rise to increased transcription and protein activation. Furthermore, a considerable number of evidence have proved the positive relation between DNA methylation and tumor occurrence and progression. On the other hand, DNA hypomethylation also contributes to oncogenesis when previously inactivated oncogenes are transcriptionally activated [17].

### 2.1. Genes Involved in Endometriosis Malignant Transformation

A number of genes, which are silenced or activated by DNA methylation, have been investigated in malignant transformation of endometriosis. Moreover, some researches that were published demonstrate the common epigenetic alteration between endometriosis and ovarian cancers. It is testified that some major genes are actually involved in the malignant transformation of ovarian endometriosis; among these contributing genes, epigenetic inactivation of Runt-related transcription factor 3 (RUNX3) [18], human mutL homolog 1 (hMLH1) [19], E-cadherin (CDH1) [20], Ras-association domain family of gene 2 (RASSF2) [21], and P16 and phosphatase and tensin homolog deleted on chromosome 10 (PTEN) [22] by promoter hypermethylation was well observed; however, long interspersed nuclear element-1 (LINE-1) [23] and syncytin-1 [24] were hypomethylated and activated. An example of this is the study carried out by Guo et al. [18] in which RUNX3 promoter hypermethylation, which results in RUNX3 inactivation and decreased RUNX3 protein expression, has been identified in the 18 of 30 (60%) patients with endometriosis-associated ovarian carcinoma (EAOC). Besides, the degree of RUNX3 hypermethylation and decreased RUNX3 protein expression in the eutopic endometrium from the EAOC group was significantly higher than that in the endometriosis (EM) and control endometrium (CE) groups. It is probable that the tissue histology in the eutopic endometrium may appear normal and intrinsic molecular abnormalities have occurred. Furthermore, it is evidenced that patients with surgical stage IC EAOC have a higher degree of RUNX3 hypermethylation than those with stages IA and IB. This phenomenon suggests that RUNX3 is implicated in the progression of malignant transformation of ovarian EM. Therefore, RUNX3 gene hypermethylation is reputed to be an early event in the pathogenesis of EAOC. Another similar study by Ren et al. [19] exploring the relationship between hMLH1 hypermethylation and malignant transformation of ovarian endometriosis is consistent with the results of the aforementioned RUNX3 promoter hypermethylation research. hMLH1 is a member of the DNA mismatch repair (MMR) system which corrects errors in DNA replication during proliferation. Ren and his colleagues illustrated this point clearly that absence of hMLH1 protein expression that resulted from aberrant promoter methylation is associated with malignant evolution of ovarian endometrium.

Other gene methylation may play an equivalent crucial role in the malignant transformation of endometriosis similar to that mentioned above, although few researchers have been able to draw on systematic researches into DNA methylation conditions of those relevant genes. It is well demonstrated that some crucial gene hypermethylation are implicated in the pathogenesis of EAOC. A recent research by Ren et al. [21] screened differentially aberrant methylated candidate genes associated with the malignant transformation of ovarian endometriosis by MCA-RDA, and nine differentially methylated candidate genes emerged in the study of malignant transformation of ovarian endometriosis. Among these nine candidate genes, RASSF2, SPOCK2, and RUNX3 were proved in other researches; therefore, the remaining six candidate genes were further studied including GSTZ1, CYP2A, GBGT1, NDUFS1, and ADAM22, as well as TRIM36. On the basis of those gene functions, they may take part in the malignant evolution of ovarian endometriosis. For example, ARID1A, identified as a tumor suppressor gene, encodes BAF250a, a key component of the SWI-SNF chromatin remodeling complex. A large number of researches demonstrate that the loss of ARID1A expression has been noted in approximately 40% of endometriotic lesions [25]. This study identified mutations in the ARID1A gene in ovarian clear cell and endometrioid carcinomas; these results represent that mutations in ARID1A are an early event in the malignant transformation of endometriosis. In a similar study, Lakshminarasimhan and his colleagues discovered that the downregulation of ARID1A expression in an endometriosis cell line enhances colony formation capacity, cell adhesiveness, and invasiveness, suggesting that low ARID1A expression might be an early event in the malignant transformation of endometriosis to ovarian clear cell carcinoma (OCCC) [26]. Although the link of ARID1A expression and OCCC transformation is well established, whether DNA methylation of ARID1A significantly matters remains elusive. In addition, a study about hypermethylation of ARID1A in breast cancer by Zhang et al. [27] demonstrated that the promoter hypermethylation in the ARID1A gene is strongly associated with ARID1A gene low mRNA expression.

### 2.2. Are Hormones Useful for Endometriosis Transformation?

It is known that endometriosis is an estrogen-sensitive and progesterone-resistant disease [28]. Estrogens have a paramount influence on various physiological processes including cell growth, reproduction, and differentiation and, in the meantime, also on pathological processes such as cancer, metabolic disease, and inflammation. The association between estrogen and various cancers is well reviewed [29]. Lots of clinical studies show that estradiol (E2) plays a key role in endometriosis. The role of E2 is regulated via the estrogen receptors (ERs) including estrogen receptors *α* (ER-*α*) and *β* (ER-*β*), which are, respectively, encoded by estrogen receptor gene 1 (ESR1) and estrogen receptor gene 2 (ESR2). Several studies investigated the expression of the ERs in the normal and ectopic endometrium of patients with endometriosis; a study reported by Cavallini et al. [30] confirmed the downregulation of ER-*α* and upregulation of ER-*β* in ovarian endometriotic tissue compared with eutopic tissue. Whether epigenetics such as DNA methylation is responsible for the expression of ERs in endometriotic cells needs to be further studied. Xue et al. [31] confirmed that the ESR2 gene promoter is hypomethylated in stromal endometriotic cells, which could be related to the upregulation of ER-*β*. However, low expression of the ER-*β* gene via promoter hypermethylation in tumors was observed [32]. Meyer et al. found [33] that ESR1 promoters (both ESR1A and ESR1B) are methylated, but the study reported by Toderow et al. [34] indicated that ER-*α* is not regulated by methylation of the promotor region in endometriosis. Without a doubt, estrogen-relevant genes and ER signal pathways are involved in the development of ovarian cancer [35–37]. Yamaguchi et al. [38], using MS-PCR to identify clear cell carcinoma-specific gene methylation, showed that 64 specific genes were involved in ER-associated pathways among the 276 hypermethylated genes. Representative ER pathway genes including ESR1, BMP4, DKK1, SOX11, SNCG, and MOSC1 are downregulated by promoter methylation, which are in accordance with decreased expression of ER-*α*. In addition, WT1, as one of the representative genes regulated by the ER-*α* signaling pathway, is downregulated in patients with endometriosis, consistent with the loss of WT1 expression in ovarian clear cell carcinoma [39, 40]. Furthermore, Akahane et al. [41] showed that decreased expression of ER-*α* occurred with progression from endometriosis to OCCC and that disappearance of hormone dependency might be associated with malignant transformation to OCCC. In conclusion, estrogen-relevant genes and pathways actually contribute to the malignant transformation of endometriosis, and the inconsistency of the ER-*β* gene expression between endometriosis and ovarian cancer and associated molecular mechanisms needs to be further investigated.

In the normal endometrium, progesterone strongly interacts with the activation of inflammatory pathways, recruits an influx of various immune cells, and mediates local inflammation [42]. Through binding to the nuclear receptors progesterone receptor isoform A (PRA) and progesterone receptor isoform B (PRB), which are members of the superfamily of ligand-activated transcription factors, the progesterone responses are regulated by directly binding to DNA and regulating the expression of target genes [43]. Several researches supported lower levels of protein expression of PRA and PRB in the eutopic endometrium and the ectopic lesions of patients compared with the normal endometrium of the control group [44]. In addition, another study, that by Fazleabas [45] using experimental animals as disease models, showed that the progesterone receptor (PR) and relevant signaling regulators exerted their effects in the early stages of endometriosis; however, with disease progression, PR expression and some targets of PR lost contact in the eutopic endometrium and the ectopic lesions of endometriosis. In short, it is well established that PR resistance plays an essential role in the occurrence, development, and progression of endometriosis, but it remains unclear whether epigenetic modifications such as DNA methylation contribute to alteration of PR-involved components. In a literature reported by Nie et al. [46], they investigated epigenetic modifications of hormones in endometriosis, and the results revealed that promoter of PRB was hypermethylated; additionally, treatment with both trichostatin A (TSA) and 5-aza-2'-deoxycytidine (ADC) increased PRB gene and protein expression in ectopic endometrial stromal cells but reduced cell viability of ectopic endometrial stromal cells. Another important study by Li et al. [47] investigating the consequences of inhibition of DNA methylation further revealed that lesion growth was ameliorated and PR and PR-target gene expression were restored; the results indicated a potential association between epigenetic regulation and PR-target signal pathways in the pathogenesis of endometriosis. In addition, one of the PR-involved components, Gata2, has been previously evidenced by Böhm et al. [48] as balancing the transcriptional activity of nuclear receptors including PGR; moreover, the expression of Gata2 gene also is consistent with that of PR in the epithelium and stroma of the uterus [49].

As indicated above, PR is dynamically associated with the occurrence of endometriosis and the progression of advanced endometriosis. Whether ovarian cancer involved in endometriosis malignant transformation also is controlled and regulated by epigenetic modification of progesterone related signaling pathways until now remains ambiguous. A previous research using immunohistochemical methods to compare the different expressions of tissues of endometriosis and EAOC indicated that the expression of PR and ER in EAOC was statistical significantly lower than that in endometriosis [50]. In addition, estrogen and progesterone regulated normal endometrial cells in proliferation and differentiation via mediating Wnt/*β*-catenin signaling whose main components include WNT7a, DKK-1, *β*-catenin, and GSK-3*β* [51]. However, abnormal activation the WNT/*β*-catenin signaling pathway via multiple regulators is reputed to be associated with ovarian cancer with epigenetic modification [52–56]. For instance, significant downregulation of the Wnt antagonist SFRP5 is observed through the promoter hypermethylation associated with overall survival in ovarian cancer; moreover, epigenetic silence of SFRP5 expression leads to activation of the Wnt pathway and promotes ovarian cancer progression [56]. As indicated above, progesterone, together with other factors, probably plays a key role in indirectly regulating the progression and development of ovarian cancer. Though elaborate mechanisms about whether hormones actually contribute to endometriosis malignant transformation, to date, still remain mysterious, we will be capable of discovering and explaining the correlation between them in the future.

### 2.3. Oxidative Stress

A large body of literature has investigated that reactive oxygen species- (ROS-) mediated oxidative stress enacts a significant role in the pathophysiology of endometriosis [57–59]. ROS are a group of oxygen including chemically reactive molecules containing superoxide (O_2_^−^), hydroxyl (OH^−^), hydrogen peroxide (H_2_O_2_), nitrogen oxide (NO), and nitrogen dioxide (NO_2_). ROS are intermediaries produced by normal oxygen metabolism, whilst during excess of ROS release, the balance between ROS and antioxidants is broken and induces cellular damage through a variety of mechanisms, finally leading to harmful effects [58]. Many theories associated with ROS-induced endometriosis progression have been elaborated so far as possibly an important factor involved in the progression of endometriosis and malignant transformation. Oxidative stress can eliminate or induce specific DNA and histone methylation by regulating corresponding enzymes such as DNMTs and ten-eleven translocations (TETs); elaborate details are reviewed by Ito et al. in a recent literature [60]. For example, a latest study carried out by Xie et al. [61] which investigated the mechanism of the correlation between oxidative stress and ARID1A gene expression illustrated that ROS decreased the expression level of ARID1A gene via regulating the methylation of its promoter. In addition, another epigenetic enzyme, the TET family of hydroxylases of encoding genes (TET1, TET2, and TET3), significantly downregulated in endometriosis [62]. TET-mediated DNA demethylation may act as a protection against oxidative stress, which also can prove the link between oxidative stress and DNA methylation of endometriosis. Moreover, oxidative stress was extensively studied and reported in the amount of mechanisms of cancer including ovarian cancer [63–67]. Oxidative stress plays an effective role in carcinogenesis through epigenetic alterations. ROS lead to tumorigenesis by inhibiting or silencing of tumor suppressor genes through promoter hypermethylation. The intestine-specific transcription factor caudal type homeobox-1 (CDX1) is downregulated with the treatment of hydrogen peroxide (H_2_O_2_) because CDX1 promoter is hypermethylated and treatment with 5-aza-dC reversed this effect [68]. In ovarian cancer, a study reported by Hou et al. [69] showed that H_2_O_2_ downregulated miR-29b by directly targeting its mRNA 3′-UTR in ovarian cancer cells. Additionally, there is a new research proved by Mahalingaiah et al. [70] which demonstrated that a low level of chronic oxidative stress results in the malignant transformation of human renal tubular epithelial cells, and the potential role is the aberrant expression of epigenetic regulatory genes involved in DNA methylation (DNMTs) as well as histone modifications (HDAC1, HAT1) in human renal tubular epithelial cells malignantly transformed by chronic oxidative stress. Given all that, oxidative stress-mediated ovarian cancer malignantly transformed by endometriosis seems to hold great promise.

## 3. Histone Modifications

Histone modification exerts an equivalent effect on epigenetic regulation the same as DNA methylation. Histones are proteins that make up nucleosomes, which are the fundamental unit of chromatin. Epigenetic modifications such as acetylation, methylation, phosphorylation, and ubiquitylation regulate chromatin structure and gene expression. Histone acetylation is mediated by a class of enzymes called histone acetyl transferases (HATs), which allow chromatin to be in a more unstable state to accomplish gene expression. In contrast, histone deacetylation is regulated by the histone deacetylases (HDACs) and converts chromatin to a more condensed or transcriptionally repressive state for inhibiting gene expression [71]. For example, acetylation at lysine 9 (K9) of H3 is implicated in the transcriptionally active condition of chromatin [16, 72]. Unlike histone acetylation, histone methylation seems to be more elusive. Histone methylation can be either stimulatory or inhibitory to the condensed state of chromatin depending on the particular lysine residue modified. Furthermore, the extent of the methylation status (mono-, di-, and trimethylation) also remains implicated. For example, trimethylation of H3K4 is involved in the transcriptionally active condition of chromatin [16, 73]; however, inverse results present in the mono-, di-, and trimethylation of H3K9, which take part in repressing gene expression [74, 75]. Besides, other types of histone modifications (phosphorylation or ubiquitination) are associated with chromatin condensation status and regulating gene expression, forming a network of sophisticated crosstalk.

### 3.1. Aberrant Enzyme Expression

There is evidence to support the theory that aberrant HDAC pathways promote cancer growth and metastasis including ovarian cancer [76–78]. HDACs play a crucial role in regulating important cell processes such as cell growth, differentiation, and apoptosis. For instance, sirtuin 1 (SIRT1) is a promising family member and a class III HDAC, which regulates histone acetylation levels as well as the DNA repair [79]. There is a study that demonstrated that SIRT1 expression was significantly increased in epithelial ovarian carcinomas (EOCs) compared to benign tumors [80]. However, another study by Xiaomeng et al. revealed that SIRT1 expression level decreased in eutopic endometrium [81]. Moreover, this is supported by a recent study indicating that the expression of nuclear HDAC1, HDAC2, and HDAC3 proteins was increased in carcinomas compared with benign tumors [77]. A study investigating HDAC expression of endometriosis showed that levels of gene and protein expressions of HDAC1 and HDAC2 were higher in ectopic endometrium than in normal endometrium [82]. Beside, another crucial histone methylation is mediated by histone methyltransferases and histone demethylase. Abnormal expressions of corresponding enzymes are probable to promote oncogene expression and progression of malignant tumors. For example, H3-K27 methylation is regulated by the enhancer of zester homolog 2 (EZH2), which is a key histone methyltransferase that belongs to a subunit of polycomb repressive complex 2. The results published by Guo et al. [83] found that EZH2 expression was significantly higher in ovarian carcinoma than in benign and normal tissues. Another study proved by Kuang and coworkers [84] revealed that EZH2 expression was positively correlated to KDM2B, which controls gene expression by the demethylation of dimethyl histone H3 lysine 36 (H3K36me2) and trimethyl histone H3 lysine 4 (H3K4me3). Both of them play an important role in the development and progression of ovarian cancer.

### 3.2. Change of Relevant Genes and Signal Pathways

In a literature, it was indicated that global histone H4 acetylation and histone H3K4 methylation level decreased significantly in both eutopic and ectopic endometrium compared with controls. However, there was no difference in H3 acetylation between endometriosis patients and controls [81]. In addition, a study using a dominant-negative histone overexpression approach demonstrated that the tumor suppressor gene RASSF1 is directly downregulated by the methylation of H3-K27. Furthermore, the results suggest that targeted epigenetic therapies of H3-K27 methylation hold great promise [85]. Furthermore, it is well recognized that signal pathways are associated with various aspects of cancer progression. A study by Hurst et al. [86] investigated the molecular mechanism of G-protein-coupled receptor (GPCR) pathways. They discovered that in the regulator of G-protein signaling 2 (RGS2), as an inhibitor of GPCRs, its protein expression is downregulated in ovarian cancer progression. A relevant study by Cacan [87] showed that loss of histone acetylation at RGS2 promoter genes results in the loss of RGS2 expression and indicated that the downregulation of the RGS2 gene is partly due to accumulation of HDACs at the promoter region of RGS2 in chemoresistant ovarian cancer cells.

As mentioned above, we talked about the association between endometriosis and ovarian cancer, such as HDAC gene expression and the increase of proteins of both HDAC1 and HDAC2 in endometriosis and ovarian cancer. Therefore, HDAC expression may be responsible for the malignant transformation of endometriosis although the association between them is still ambiguous. On the other hand, more marked differences were observed in the results of two kinds of researches, such as SIRT1 upregulation in ovarian cancer and downregulation in endometriosis. In spite of those opposite results, the mechanisms of endometriosis malignant evolution will be completed in the future.

### 3.3. Inflammation and Immune Disorders Need to Be Further Investigated in Epigenetic Modification

Without a doubt, endometriosis is a complex, chronic inflammatory disease with variable symptoms in women. Inflammation and immune disorders absolutely play a key role in the pathobiology of endometriosis; therefore, inflammatory cells and inflammatory cytokines are regulated as target components in endometriosis patients; also, the immune system of women with endometriosis is also dysfunctional. The immune system contains a variety of immune cells, including macrophages, dendritic cells, natural killer cells, T helper cells, and B cells, which have been proved to be disordered in patients with endometriosis [88, 89]. Disorders of inflammatory cell populations in ectopic endometrium and their secretory products exert a harmful influence on normal microenvironment, inducing the development of the disease. Moreover, the immune system and endometrial cells secrete several cytokines and growth factors that promote invasion and growth of ectopic endometrium [90]; those cytokines and growth factors are representative inflammatory mediators, leading to inflammatory response and finally aggravating endometriosis, even causing ovarian cancer.

**Figure 1:**
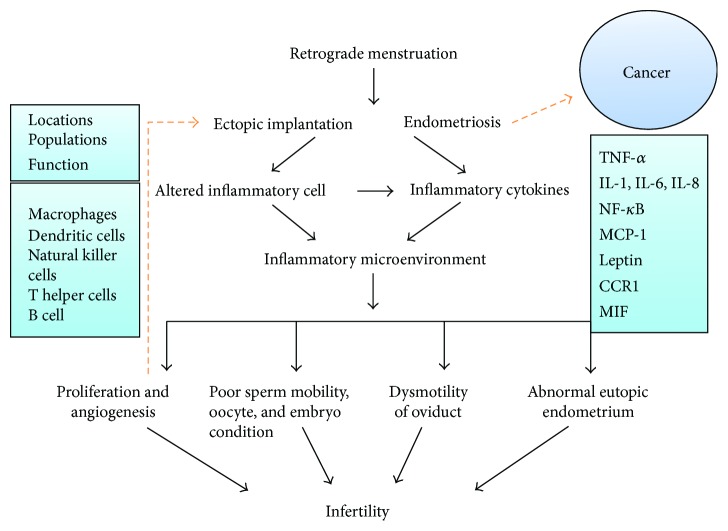
The potential inflammatory mechanisms between endometriosis and infertility. The figure indicates that various factors may result in infertility in patients with endometriosis. Inflammatory cytokines are secreted by inflammatory cells including TNF-*α*, IL-1, IL-6, IL-8, NF-*κ*B, MCP-1, leptin, CCR1, MIF, and COX-2. Inflammatory responses depend on locations, populations, and functions of inflammatory cells, which include macrophages, dendritic cells, natural killer cells, T helper cells, and B cells. On the one hand, inflammatory responses alter microenvironment and influence various aspects of fertility; on the other hand, chronic exposure to microenvironments with overproduced inflammatory factors leads to ectopic implant proliferations and angiogenesis, which in turn promote growth and invasion of ectopic endometrium even in the development of cancer.

To date, the most robust pathogenic hypothesis involved in inflammation response is based on the so-called retrograde menstruation phenomenon. Through retrograde flow, viable endometrial fragments reach and implant onto the peritoneum and abdominal organs, leading to chronic inflammation with formation of adhesions and severe infertility. Chronic inflammation, in turn, also promotes proliferation and growth of ectopic endometrial tissue [28]. The presence of ectopic tissue is associated with secretion disorder of inflammatory cells and factors. Macrophages and associated signaling cascade are alternatively activated in patients with endometriosis, which were observed in the study reported by Mahdian et al. [91]. In addition, various inflammatory factors play different roles in infertility in patients with endometriosis [92, 93]. A study by Yang et al. [94] investigated the relations between exposure to pelvic microenvironments with overproduced inflammatory factors and structural or functional tissue abnormalities; their data showed that telocytes (TCs; interstitial Cajal-like cells (ICLCs)) were significantly decreased and interstitial fibrosis was observed, accompanied with an increased level of inducible nitric oxide synthase (iNOS), cyclooxygenase-2 (COX-2), lipid peroxide (LPO), and estradiol, which suggested that inflammation induced TC damage and fibrosis and dysmotility of the oviduct finally leading to subfertility or infertility. Yoshida et al. [95] indicated that interleukins 1 and 6 directly affect sperm mobility. In addition, Hosseini et al. indicated that epigenetic changes of CYP19A1 (aromatase) gene promoters may lead to poor oocyte and embryo condition by impairing follicular steroidogenesis in patients with endometriosis [96]. Tao et al., who studied the pathogenesis of endometriosis-associated infertility, confirmed the tight correlation between monocyte chemotactic protein- (MCP-) 1 and peritoneal leptin levels and infertility in the early stage of endometriosis [97]. Rathore et al. indicated that ghrelin and leptin might contribute to the pathophysiology of infertility, and leptin is associated with inflammatory factors such as IL-6 in patients with endometriosis [98] (Figure 1).

In recent years, cytokines caught the intense attention of researchers due to their involvement in the pathogenesis of endometriosis and cancer. Endometriosis is often accompanied by marked changes of inflammatory cytokines, including epithelial cell-derived neutrophil-activating peptide-78 (ENA-78), macrophage migration inhibitory factor (MIF), high-sensitivity C-reactive protein (hs-CRP), tumor necrosis factors (TNF-*α*), interleukin-1*β* (IL-1*β*), IL-6, IL-8, interferon-induced protein 10 (IP-10), and chemokine receptor 1 (CCR1) [99, 100]. There is great expectations for one of them, IL-8, in the development and progression of endometriosis. The literature strongly suggests that IL-8 might play an important role in adhesion and growth of the endometrial implants [101, 102]. A literature by Ulukus et al. [103] showed higher epithelial IL-8 expression in eutopic endometrium of patients with endometriosis, as compared to normal women. In addition, the literature suggested that increased IL-8 expression levels in women with endometriosis might contribute to the development of endometriosis and finally progression of chronic inflammation, even probably malignant transformation [104, 105]. In ovarian cancer, epigenetic modifications are regarded as regulations and mediations of ovarian cancer development, and epigenetic therapies as inhibition of ovarian cancer cells. For instance, the study investigated the specific involvement of HDACs and HATs in the epigenetic regulation of IL-8 expression in ovarian cancer cells; the results indicated that the IL-8 expression in OC cells is regulated by CBP and might enhance effectiveness of HDAC inhibitors in OC treatment [106]. They previously showed that inhibition of histone deacetylase (HDAC) activity increased IL-8 expression in OC cells, resulting in their increased survival and proliferation [107].

The role of chemokine epigenetic regulations in endometriosis malignant transformation has so far remained ambiguous, but those researches suggest that chemokines might appear as a valuable tool to influence the correlation between endometriosis and ovarian cancers and perform early diagnosis of ovarian tumors.

## 4. MicroRNA Alteration

Over the past 20 years, another epigenetic regulation of gene expression has been discovered and well established, which participates in posttranscriptional gene downregulation mediated by small, non-protein-coding RNA molecules named microRNAs (miRNAs) [108, 109]. Since their initial discovery in 1993 [110], small noncoding RNAs or microRNAs have been intensely investigated across almost all biomedical fields including tumors. MicroRNAs, a class of single strand, are endogenously expressed, and noncoding RNAs, approximately 22 nucleotides in length, were found to mediate a series of essential biological processes including cell cycle, differentiation, development, and apoptosis as well as metabolism [111–114]. Plenty of work has been absorbed in investigating the biogenesis of miRNAs.

miRNA host gene is transcribed by RNA polymerase II, and transcription products are so-called pri-miRNA [115]. The pri-miRNA has to undergo two processing steps in order to become a mature miRNA. First, pri-miRNA is precisely recognized and cleaved by the enzyme Drosha which interacted with an RNA binding protein DGCR8, forming the so-called “microprocessor.” The following processing step occurs in cytoplasm: the predominant enzyme called Dicer, also associated with an RNA binding protein named TRBP [116], cleaves the pri-miRNA to a short RNA duplex approximately 21–25 nucleotides in length, depending on the type of Dicer and miRNA [117]. The two strands of the duplex have their own independent influence: one strand of duplex is incorporated into RNA-induced silencing complex (RISC) as a mature miRNA exerting its function, and the remaining strand is usually degraded. However, both strands of some miRNAs are likely to be selected into RISC. The RISC or microRNAs are able to prevent mRNA translation and induce mRNA degradation by matching 3′ untranslated regions of target mRNAs; this phenomenon is known as RNA interference. MicroRNAs are strictly controlled in normal cells. Once microRNAs become deregulated, those aberrant productions can lead to the occurrence and progression of diseases. Researches showed that microRNAs may be associated with the pathogenesis of various human cancers.

### 4.1. Contributing MicroRNAs in Ovarian Cancer and Endometriosis

In ovarian cancer, the role of miRNAs is present in different biological processes including cell cycle, apoptosis, proliferation, invasion, and metastasis, and even chemoresistance. miRNAs are mediated by Drosha and Dicer; several reports showed that Dicer and Drosha mRNA expression levels and the corresponding proteins were decreased in the majority of ovarian cancers compared with normal tissues [118, 119]. Moreover, ovarian cancers were found to significantly upregulate four members of miR-200 family of miRNAs containing miR-200a, miR-141, miR-200c, and miR-200b, whereas miR-199a, miR-140, miR-145, and miR-125b1 were downregulated among most miRNAs [120]. On the other hand, alterations in the expression levels of different members of miR-200 family are differently associated with the distinct histotypes of ovarian carcinomas. miR-200a and miR-200c overexpressions occur in all the three histotypes including serous and endometrioid as well as clear cell, whereas miR-200b and miR-141 upmodulation exists in endometrioid and serous histotypes [120]. Additionally, another family of miRNAs, the let-7 (lethal-7) family, as tumor suppressor miRNAs, also gets widespread attention in multiple human tumors [121]. Remarkably reduced expressions of the let-7i were observed in tumors of cancer patients with poor survival [122, 123]. In the ovarian cancer, let-7i significantly reduced expression in chemotherapy-resistant patients as reported in a study; moreover, reduced let-7i expression significantly increased the resistance of ovarian cancer cells to the chemotherapy drug *cis*-platinum [123]. Several studies also unravel other miRNA expressions associated with ovarian cancers; for instance, miR-16, miR-20a, miR-21, miR-23a, miR-23b, miR-27a, miR-93, miR-30c and miR-30d, and miR-30e-3p were found to be overregulated, whereas miR-10b, miR-26a, miR-29a, miR-99a, miR-100, miR-125a, miR-125b, miR-143, miR-145, miR-199a, miR-214, miR-22, and miR-519a have opposite expressions [124–126]. Consequently, different miRNAs exert their influence on ovarian cancer development and progression.

Only few researches about malignant transformation were published. Several recent studies investigated the mechanisms of malignant transformation of endometriosis. Tissue inhibitor of metalloproteinases 3 (TIMP3), a proapoptotic protein [127], is proved by Qin and coworkers as a direct target of miR-191 [128]. The data of the study revealed that miR-191 expression was significantly higher in both endometriosis and EAOC and that TIMP3 expression was negatively correlated with miR-191 expression [129]. Additionally, another study, discussing whether cancer-associated miRNA single nucleotide polymorphisms (miRSNPs) accelerate endometriosis development and progression, demonstrated that MIR196A2 and MIR100 influenced endometriosis development and related clinical phenotypes [130].

### 4.2. EMT Affects Early Stage of the Oncogenic Transformation

Epithelial-mesenchymal transition (EMT) is a highly conserved cellular process that converts immotile and polarized epithelial cells to motile mesenchymal cells, which occurs in embryonic development, fibrosis, and wound healing; meantime, cancer development and progression that resemble embryonic development are regulated and controlled by EMT [131]. As usual, whether EMT occurs or not is detected by corresponding protein expression marks; E-cadherin and cytokeratins are the most common markers for the epithelial phenotypes and N-cadherin and vimentin for the mesenchymal [131]. Multiple noncoding RNAs are reputed to govern EMT; two major regulatory networks are demonstrated to be considered as the core regulatory machinery—the miR-34-SNAI1 and miR-200-ZEB1 axes—which are also controlled by various mediators.

EMT processes enhance migration and invasion of cells, which are prerequisites for the implantation of endometriotic lesions. A previous study reported by Bartley et al. [132] showed that the expression levels of N-cadherin, Twist and Snail, were significantly higher in endometriosis than in endometrium. However, in endometriosis, the expression of E-cadherin was inversely decreased in comparison with that in endometrium. Another study also proved that EMT-related processes might be involved in the pathogenesis of pelvic endometriosis [133]. Filigheddu et al. [134] showed the downregulation of miR-200b expression in the ectopic endometrium compared with the eutopic endometrium of endometriosis patients, together with enhancing of EMT. Eggers et al. [135], who investigated whether miR-200b expression contributes to EMT and invasive growth in endometriosis, indicated that upregulation of miR-200b reverts EMT and inhibits migration and invasion of cells of the endometriotic cell.

The significance of EMT during cancer progression has been commonly recognized, and EMT processes are thought to take part in many cancer cell metastases and progression. For instance, EMT occurs at the invasive front and single mesenchymal-like cells are detected to lose E-cadherin expression in colon carcinoma [136]. Knockdown of Linc-ROR in ovarian cancer cell lines prevents EMT processes through the repression of Wnt/*β*-catenin signaling; the results suggest that EMT can be an important phenomenon in the invasion and metastasis of ovarian cancer [137]. Another study showed that inhibition of miR-23a reduced the TGF-*β*1-induced EMT, invasion, and metastasis in breast cancer cells though directly targeting CDH1 that activated induced Wnt/*β*-catenin signaling [138]. This phenomenon is also discovered in other tumors, which reveals an EMT expression profile and shows increased vimentin and loss of E-cadherin.

As mentioned above, EMT is reputed to be an invasive behavior and enables normal cells to be more aggressive, further having a potential malignant tendency. EMT may exert its momentous transitional effects on endometriosis malignant transformation; furthermore, the occurrence of EMT is probable in an early stage event of endometriosis malignant ovarian cancer in the future.

## 5. Conclusion

The overall aim of this review was to summarize the epigenetic modifications of the relations between endometriosis malignant transformation and ovarian cancer; moreover, due to the constraints of research progress and attention, we talked more about the potential correlations between them by relevant literatures. Beyond the abovementioned epigenetic modifications, other epigenetics such as long noncoding RNA (lncRNA) and posttranslational modifications (PTMs) are associated with the pathogenesis of endometriosis and ovarian cancer [139–143]. As complex gynecologic disease is closely related to the cancer, enigmatic etiology of endometriosis and mechanism of endometriosis malignant transformation are absolute worth correctly uncovering and elucidating in the future. In recent years, researchers have made significant strides in understanding the disease-specific molecular pathways governing the development of endometriosis in ectopic locations by studying the blood, peritoneal fluid, and eutopic endometrium of women with the disease [144–147]. Vicente-Muñoz et al. [147] identified the plasma metabolites of endometriosis patients and found higher concentration of valine, fucose, choline-containing metabolites, lysine/arginine, and lipoproteins and lower concentration of creatinine than in healthy women, which can help to get a better understanding of the molecular mechanisms of endometriosis. Studying epigenetic modifications of endometriosis, as well as investigating the correlation between endometriosis and ovarian cancer, will propel our understanding of the pathogenesis of endometriosis malignant transformation, with the potential for early diagnostic interventions and new effective therapies.
